# Supplementary material for: Exploring the Genomic Landscape of Hepatobiliary Cancers to Establish a Novel Molecular Classification System
Source: Cancers (Basel). 2024 Jan 11;16(2):325. doi: 10.3390/cancers16020325 (PMC10814719; doi:10.3390/cancers16020325)

Supplemental Figures

***Supplemental Figure S1.** The Genomic Landscape of Tissue of Origin Subtypes

Hepatobiliary cancer (HBC) genomic landscaping results (n=329) identified differences in mutated genes between tissue of origin subtypes. *IDH1* and *KRAS* mutations were associated with CCA, *CTNNB1* and *TERT* mutations with HCC, and all HBCs had *TP53* and *ARID1* mutations (p<0.001).

*Analysis performed prior to the creation of hepatobiliary cancer molecular subtypes

***Supplemental Figure S1a.** Cholangiocarcinoma (CCA) most common mutated genes.

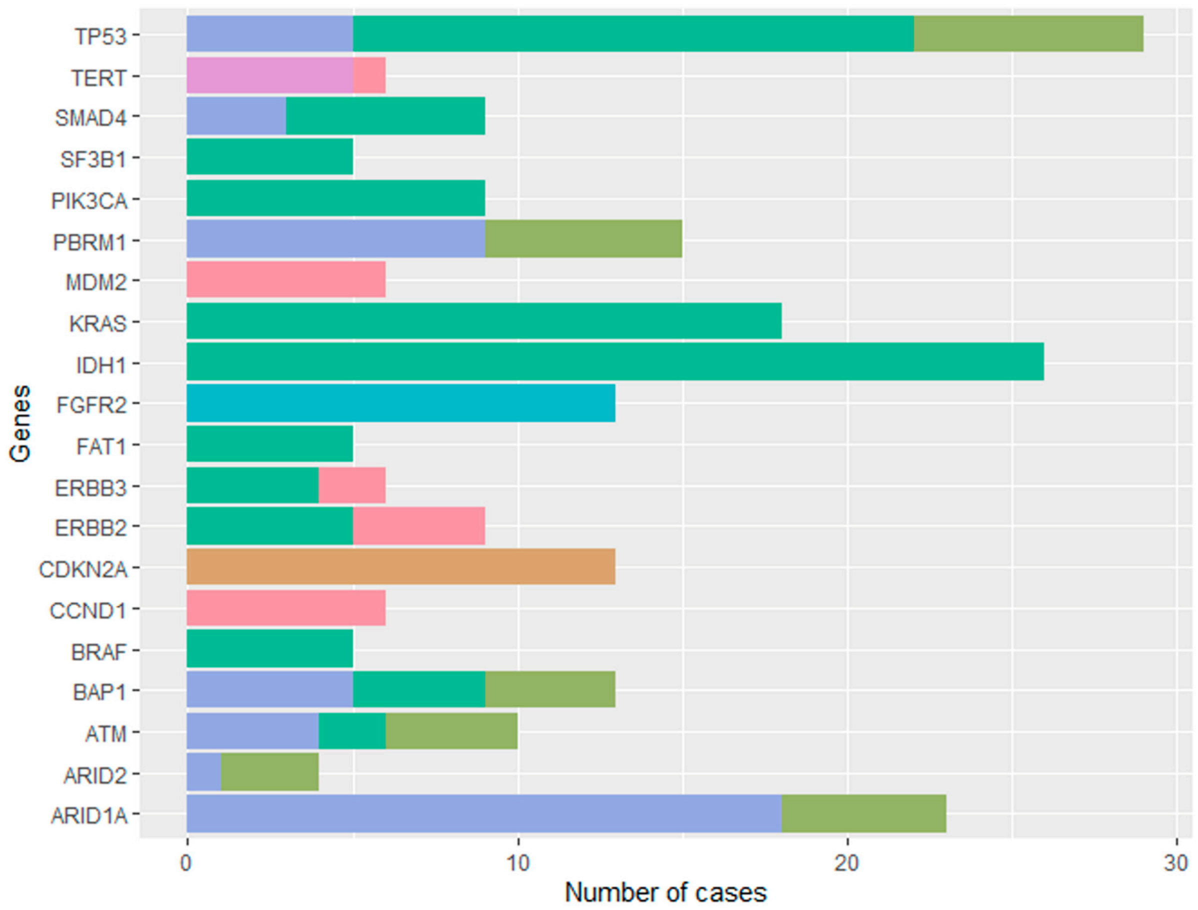

*__Supplemental Figure S1b.__ Hepatocellular carcinoma most common mutated genes.

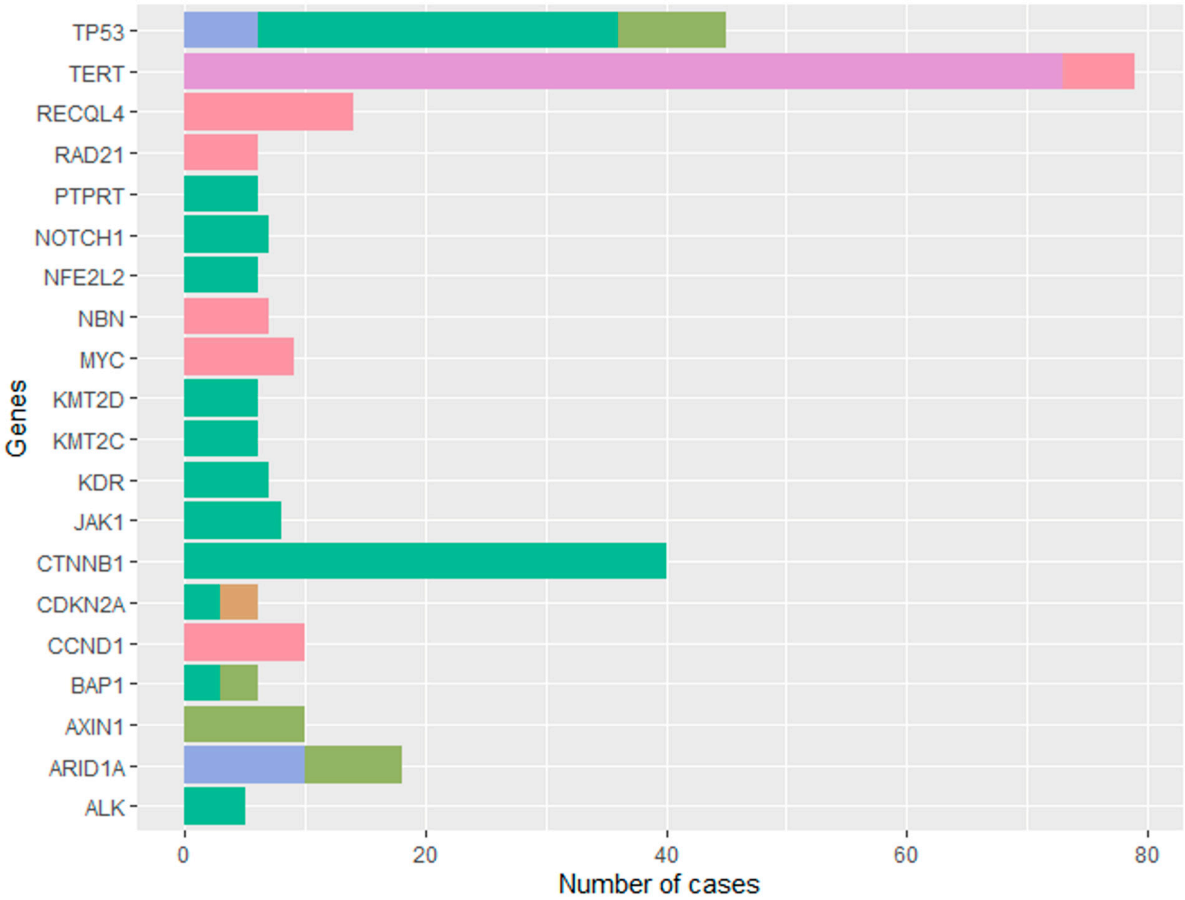

*__Supplemental Figure S1c.__ Gallbladder Carcinoma most common mutated genes.

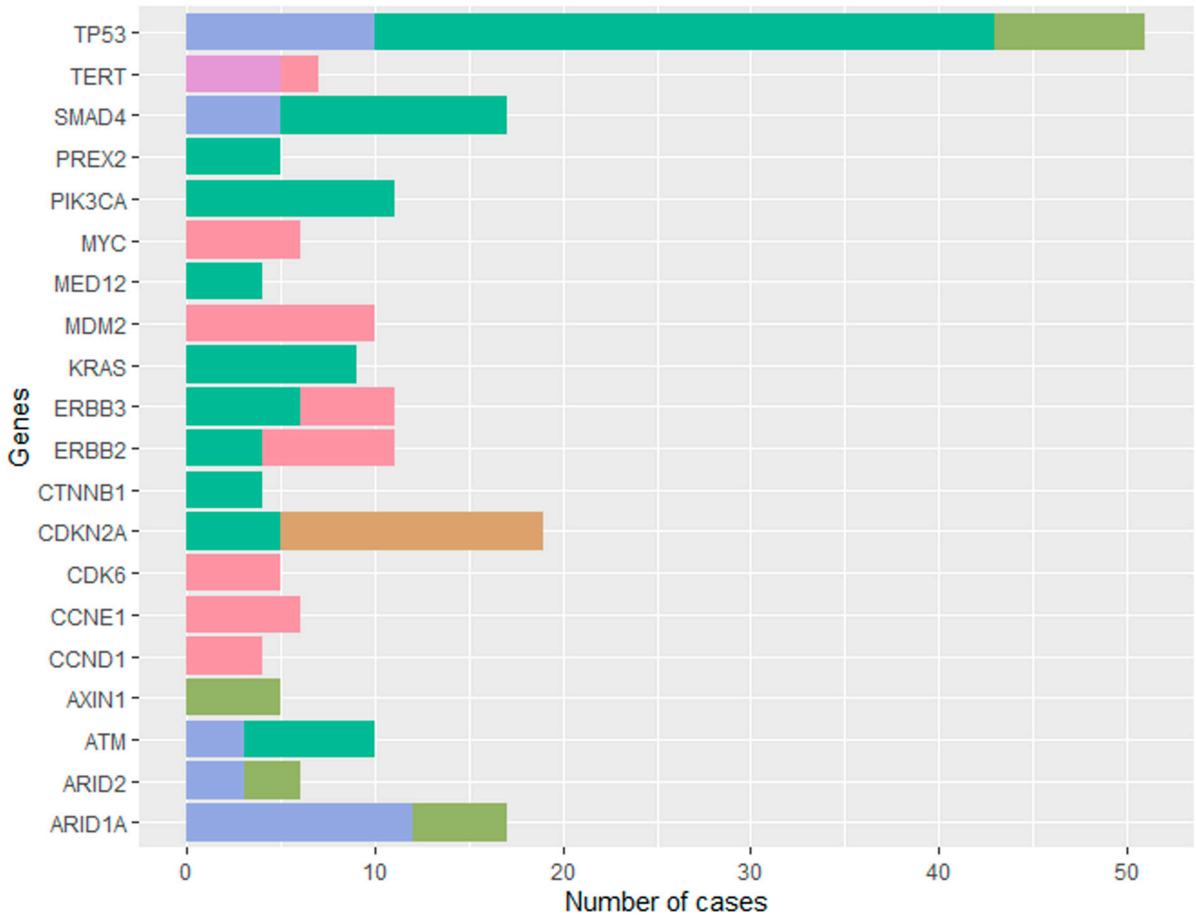

Supplement: Supplementary file 1 [file cancers-16-00325-s001.zip › cancers-2752501-supplementary.pdf]
